# Unraveling the complexities of caffeine: metabolism, genetics, evolution, and health

**DOI:** 10.1186/s41065-026-00648-z

**Published:** 2026-02-02

**Authors:** Xinyi Liu, Shuhua Xu

**Affiliations:** Center for Evolutionary Biology, School of Life Sciences, Fudan University, Shanghai, 200438 China

**Keywords:** Caffeine, Pharmacogenomics, Evolutionary genetics, *CYP1A2*, Cardiovascular health, Tea versus coffee

## Abstract

Caffeine (1,3,7-trimethylxanthine) is the most widely consumed psychoactive substance globally, yet individuals differ strikingly in their physiological and health responses. These differences arise from the combined effects of genetic variation, evolutionary history, lifestyle exposures, and cultural practices. In this review, we synthesize current knowledge on the pharmacokinetics of caffeine, emphasizing the central role of the hepatic enzyme CYP1A2 and its transcriptional regulator AHR in determining metabolic rate. We summarize functional polymorphisms, haplotypes, and genome-wide association signals that underlie the spectrum from slow to rapid metabolizers, and integrate population-genetic and ancient DNA evidence to reveal how evolutionary pressures have shaped global patterns of caffeine tolerance. We further examine non-genetic modulators—including smoking, pregnancy, diet, and commonly prescribed medications—that dynamically influence CYP1A2 activity. Finally, we discuss how genetic variation interacts with coffee and tea’s diverse bioactive components to influence cardiovascular, neurological, and metabolic outcomes. By uniting pharmacogenomics, evolutionary genetics, and epidemiology, this review highlights the need for personalized caffeine intake recommendations that account for genetic background, beverage preparation methods, and individual health status.

## Introduction

**Fig. 1:**
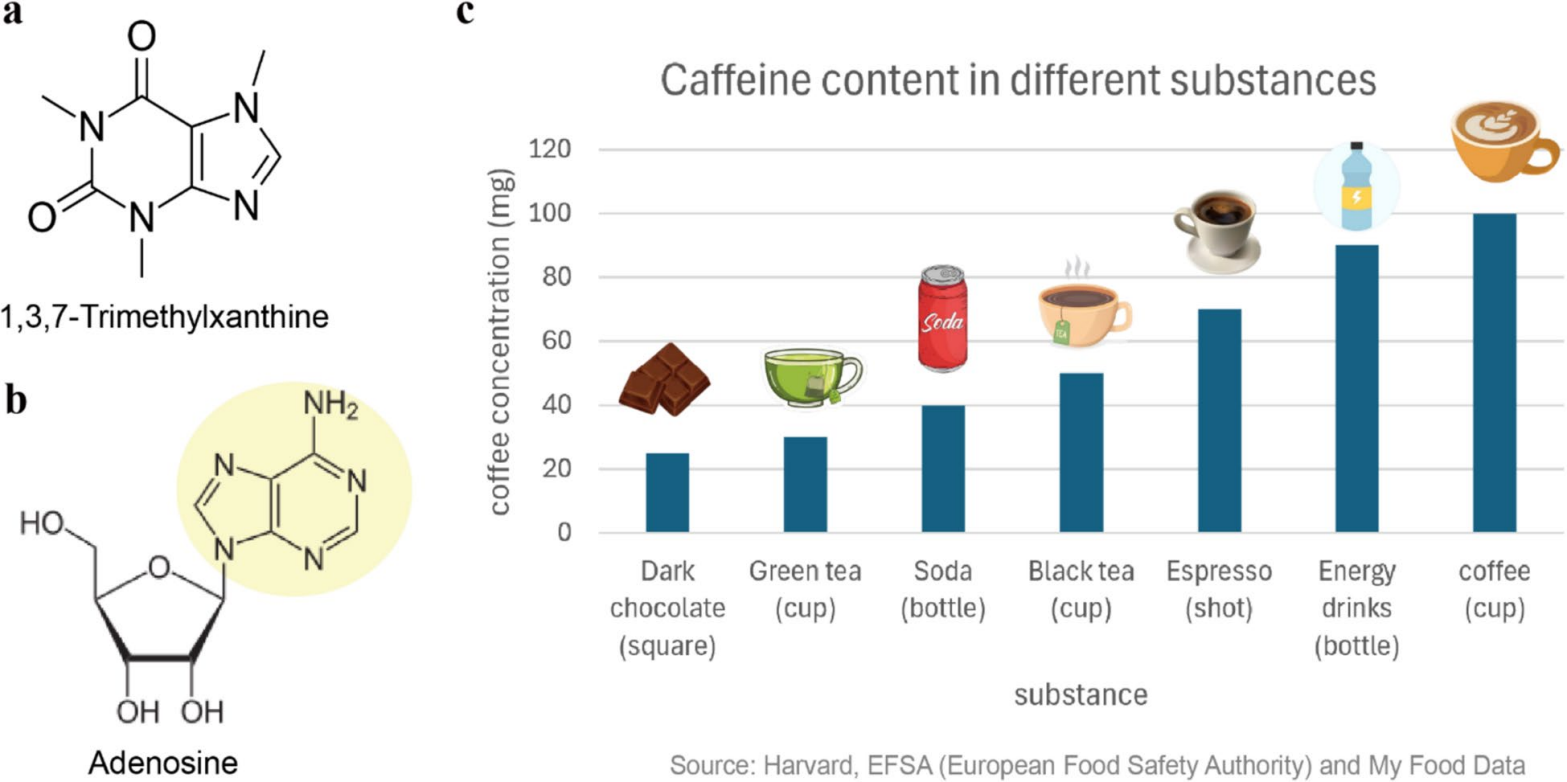
Structure, properties, and natural abundance of caffeine. **a** Chemical structure of caffeine; **b** Chemical structure of adenosine, with the yellow highlight indicating structures similar to caffeine; **c** Caffeine content in various natural sources, including coffee, tea, cocoa, and other beverages. Data sourced from Harvard, EFSA and My Food Data

Caffeine (1,3,7-trimethylxanthine) is a purine alkaloid and the most widely consumed psychoactive substance globally (Fig. 1a). Large-scale surveys indicate that the worldwide consumption of caffeine has been 120 K tons per year approximately [11]. This widespread consumption is not merely a cultural habit but is also driven by caffeine's capacity to mitigate fatigue and improve focus. The basis for this effect lies in its structural similarity to adenosine, an endogenous neuromodulator in the brain (Fig. 1b). Caffeine acts as a competitive antagonist at adenosine receptors (primarily A₁ and A₂A subtypes) in the central nervous system [100]. While A1 receptors promote sleep and reduce neuronal excitability, A2A receptors modulate mood and locomotion by interacting with dopaminergic pathways [32, 92]. By blocking both receptor types, caffeine can promote alertness and enhance mood or motor performance [36]. Caffeine is commonly present in plant-based sources such as coffee beans, tea leaves, cocoa, and kola nuts. In addition to its natural sources, synthetic caffeine also appears as an additive in soft drinks and energy beverages (Fig. 1c) for its stimulant effects [19, 81].

However, despite its universal consumption, individuals exhibit striking variability in their responses to caffeine—ranging from heightened alertness and improved performance to debilitating anxiety, insomnia, and varied cardiovascular effects. A critical question thus arises: What are the underlying determinants of such vast individual variability in caffeine sensitivity and tolerance?

The answer to this question lies not in a single factor but in a complex interplay of intrinsic and extrinsic influences spanning from an individual's genetic blueprint to the intake pattern. This variability is primarily orchestrated by: (1) Genetic polymorphisms governing the activity of key metabolic enzymes and target receptors; (2) Environmental and cultural modulators, including diet, smoking, medication, and habitual intake patterns; (3) Evolutionary pressures that have shaped these genetic variations across different human populations. The complexity of this genetic architecture was recently underscored by a systematic review, which synthesized evidence from over 1.85 million individuals, confirming the roles of key genes like *CYP1A2* and *AHR,* and further highlighting the polygenic nature of caffeine intake, involving both metabolic and reward pathways [77]. This review is structured to unravel this complexity by first delineating the pharmacokinetic journey of caffeine, with a focus on its primary metabolic enzyme, CYP1A2. We then explore the genetic and evolutionary origins of population-level differences in caffeine metabolism, integrating novel insights from genome-wide association studies (GWAS) and ancient DNA analysis. Furthermore, we examine how environmental factors modulate these innate predispositions. Finally, we synthesize these perspectives to discuss the personalized health implications of caffeine consumption as well as tea consumption as an alternative, with a focus on how genetic polymorphisms defining fast and slow metabolizer status can inform individual risk–benefit profiles.

### Absorption, metabolism, and elimination of caffeine

The immense individual variability in caffeine response is fundamentally rooted in differences in its pharmacokinetics—the process by which the body absorbs, distributes, metabolizes, and excretes the compound. Understanding this process stepwise is crucial to appreciating the genetic and environmental factors that modulate it.

Following oral consumption, caffeine is rapidly and almost completely absorbed from the gastrointestinal tract, primarily in the small intestine, with peak plasma concentrations typically occurring within 30 to 60 min [16]. The absorption rate constant (K₀₁) is approximately 0.33 per minute, leading to 99% bioavailability within about 45 min, with no significant first-pass effect [3, 118]. Though water-soluble, its hydrophobic properties facilitate efficient passive diffusion across biological membranes [68, 69]. Once absorbed, caffeine is evenly distributed in all body fluids such as blood plasma including umbilical cord blood, cerebrospinal fluid, saliva, bile, semen, breast milk, and all organs [16]. The volume of distribution of caffeine ranges between 0.5 and 0.75 L/kg [3], reflecting widespread tissue penetration. Notably, its small molecular size and lipophilicity enable efficient crossing of the blood–brain barrier via simple diffusion and carrier-mediated transport [80], thereby exerting its central nervous system effects (Table 1).

**Table 1:** Key pharmacokinetic parameters of caffeine

Parameter	Value	Interpretation	Reference
Absorption Rate (K₀₁)	~ 0.33 min⁻^1^	Rapid and complete absorption from the gastrointestinal tract	[3]
Volume of Distribution (Vd)	0.5—0.75 L/kg	Widespread distribution into all tissues and body fluids	[3]
Time to Peak Concentration (Tₘₐₓ)	30—60 min	The time after oral ingestion when plasma concentration peaks	[16]
Half-Life (t₁/₂)	~ 4 h	The time required for plasma concentration to reduce by 50%	[57]
Clearance (CL)	1.5 ± 0.7 mL·kg⁻^1^·min⁻^1^	The primary metric of metabolic efficiency	[14]

The systemic clearance of caffeine is dominated by hepatic metabolism, with only about 0.5% to 3.0% of a dose excreted unchanged by the kidneys [112]. In the liver, caffeine is primarily catalyzed by the cytochrome P450 monooxygenase enzyme CYP1A2 [89]. This enzyme accounts for approximately 13–15% of the total cytochrome P450 content in human liver and is responsible for over 90% of the initial metabolic steps of caffeine clearance [5]. CYP1A2 catalyzes the biotransformation of caffeine through successive demethylation reactions, leading to the formation of three primary dimethylxanthine metabolites: paraxanthine (1,7-dimethylxanthine), theobromine (3,7-dimethylxanthine), and theophylline (1,3-dimethylxanthine). Paraxanthine promotes lipolysis, leading to elevated plasma glycerol and free fatty acid levels [13]. Theobromine, predominantly found in cocoa, acts as a vasodilator and diuretic [107]. Theophylline, known for its ability to relax bronchial smooth muscle, is therapeutically used in asthma management [10]. The regioselectivity of CYP1A2-mediated metabolism favors N3-demethylation, forming paraxanthine, which constitutes over 80% of the metabolic pathway [89]. This is followed by N1-demethylation (yielding theobromine, ~ 10–12%) and N7-demethylation (producing theophylline, ~ 4%). CYP1A2 interacts with caffeine via four distinct binding modes, corresponding to its metabolic sites, with the N3-site demonstrating a predominant binding affinity that explains its role as the primary metabolic route [46].

Although CYP1A2 is the principal enzyme involved, other cytochrome P450 isoforms contribute to minor pathways. For example, CYP2E1 participates in the demethylation pathways, particularly toward theophylline and theobromine formation, while CYP3A4 mediate C8-hydroxylation to form 1,3,7-trimethyluric acid [49, 68, 69]. Nevertheless, the relative contribution of these isoforms is markedly lower than that of CYP1A2.

Following the initial N-demethylation, the primary dimethylxanthine metabolites undergo further Phase I reactions, primarily mediated by various CYP isoforms. Paraxanthine is metabolized via CYP1A2, CYP2A6 into secondary metabolites, including 1-methylxanthine and 1,7-dimethyluric acid. Theobromine and theophylline are similarly processed through additional demethylation and oxidation steps, often involving xanthine oxidase [89]. Paraxanthine also undergoes Phase II conjugation through acetylation, a reaction that generates 5-acetylamino-6-formylamino-3-methyluracil (AFMU) as a key metabolite [12].

The pronounced interindividual variability in CYP1A2 enzyme activity, which can vary up to 15-fold in the human population, translates into a broad spectrum of physiological outcomes [40]. This variability is not random,it is systematically governed by a complex interplay of intrinsic and extrinsic factors.

Primarily, CYP1A2 activity is regulated at the genetic level. Polymorphisms within the *CYP1A2* gene and its regulatory regions determine an individual's innate metabolic capacity, creating a population continuum from "slow" to "rapid" metabolizers [52, 67]. The following section will delve into the genetic architecture underlying this variation, exploring how hereditary factors have shaped global patterns of caffeine metabolism and tolerance.

**Fig. 2:**
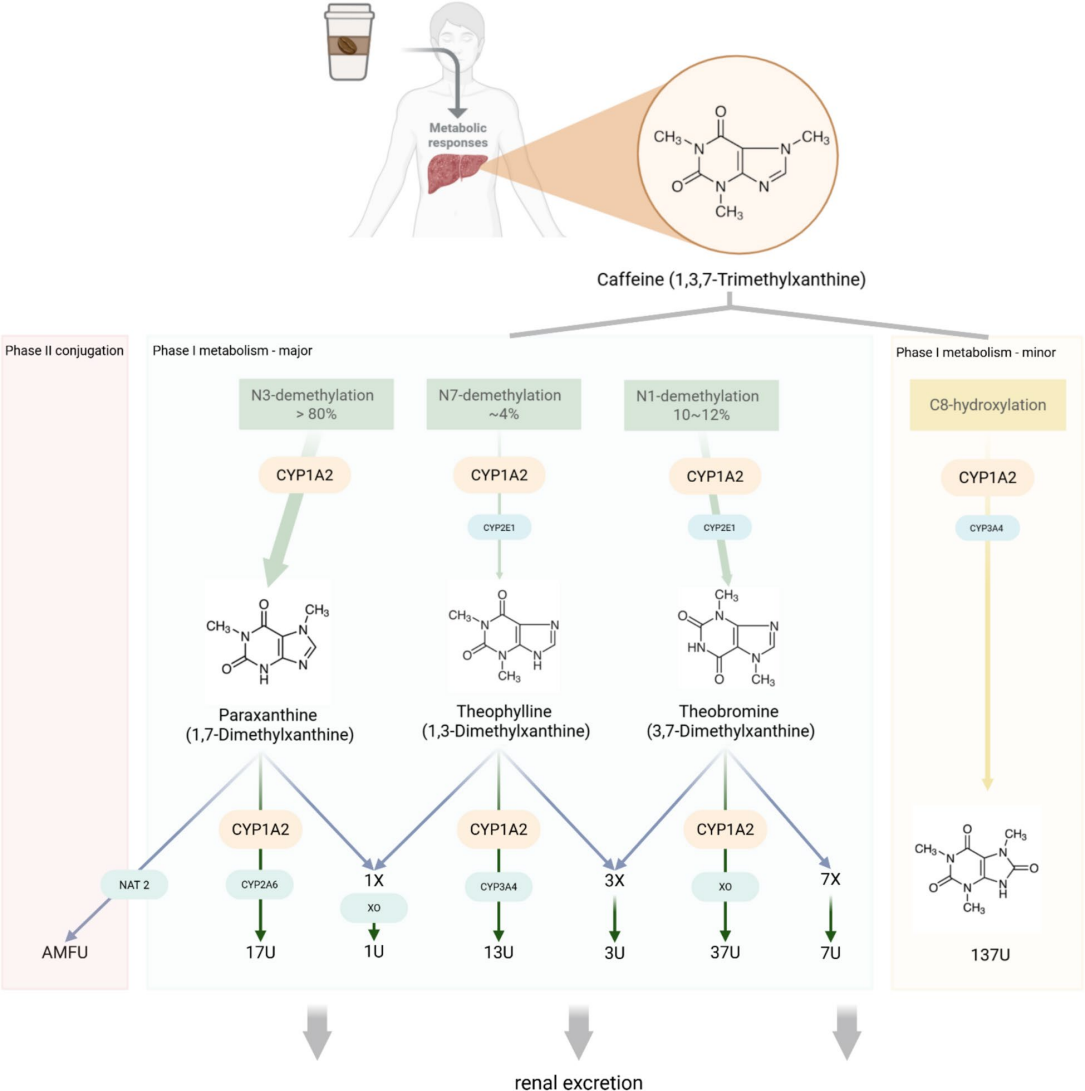
Caffeine metabolism pathways. 17U: 1,7-dimethyluric acid; 37U: 3,7-dimethyluric acid; 13U: 1,3-dimethyluric acid; 1U: 1-methyluric acid; 3U: 3-methyluric acid; 7U: 7-methyluric acid; 137U: 1,3,7-trimethyluric acid, NAT2: N-acetyltransferase 2, AMFU: 5-acetylamino-6-formylamino-3-methyluracil

While this genetic predisposition provides the foundational blueprint, it is not the sole determinant. As will be discussed in subsequent sections, environmental exposures and physiological states can profoundly modulate enzyme activity, creating a dynamic interplay between one's genetic makeup and lived experience that ultimately defines the individual response to caffeine Fig. 2.

### The genetic and evolutionary foundations of caffeine tolerance

The interindividual variability in caffeine metabolism is profoundly influenced by genetic polymorphisms within the *CYP1A2* gene. The human *CYP1A2* gene is located on chromosome 15q24.1, spans approximately 7.8 kb, and comprises seven exons and six introns [56]. It forms a cluster with the related *CYP1A1* gene, sharing a common 5´-flanking region, which suggests coordinated regulation of these two key detoxification genes [33]. To ensure clarity, all genetic analyses in this review are based on the canonical transcript (MANE Select) for the key genes discussed unless otherwise stated. For the *CYP1A2* gene, we refer to transcript NM_000761.5, and for the AHR gene, transcript NM_001621.4.

Research over the past two decades has identified several key single-nucleotide polymorphisms (SNPs) that functionally alter CYP1A2 enzyme activity. These SNPs form the basis of common defined haplotypes. The CYP1A2*1A haplotype is considered as the wild-type reference. The CYP1A2*1C haplotype (defined by − 3860 G > A, rs2069514) and the CYP1A2*1 K haplotype (defined by − 739 T > G, rs2069526 and − 729 C > T, rs12720461) are consistently associated with decreased CYP1A2 enzyme activity in vivo. In contrast, the CYP1A2*1F haplotype (defined by − 163 C > A, rs762551) is generally associated with increased inducibility, leading to a "hyper-inducible" phenotype upon exposure to environmental or dietary stimuli such as smoking [97, 114] (Table 2).

**Table 2:** Key CYP1A2 haplotypes and their functional impact on enzyme activity

Variant	Haplotype	Association	Cases	*P*-Value	Reference
rs2069514	CYP1A2*1C	Genotypes AA + AG are associated with decreased metabolism of caffeine as compared to genotype GG	50	< 0.05	[85]
rs762551	CYP1A2*1F	Genotypes AA + AC is associated with increased metabolism of caffeine as compared to genotype CC	414	= 0.001	[67]
rs762551	CYP1A2*1F	Genotype AA is associated with increased metabolism of caffeine in people with Tobacco Use Disorder as compared to genotypes AC + CC	35	= 0.04	[41]
rs12720461	CYP1A2*1 K	Genotype CT is associated with decreased metabolism of caffeine in healthy individuals as compared to genotype CC	173	< 0.02	[2]

This genetic architecture translates directly into the "slow" and "rapid" metabolizer phenotypes. These phenotypic differences are quantitatively measured using the paraxanthine-to-caffeine metabolic ratio (17X/137X) in plasma or saliva, which serves as a well-validated biomarker for in vivo CYP1A2 activity [21] [115]. Notably, the − 163 C > A polymorphism (rs762551) significantly influences this metabolic ratio specifically under induction conditions. In heavy coffee consumers, individuals with the A/A genotype exhibit significantly higher 17X/137X ratios compared to C/A and C/C carriers, with this − 163 C > A polymorphism accounting for 14% and 22% of the interindividual variability in CYP1A2 activity among Serbian and Swedish heavy coffee consumers, respectively [38]. Thus, common genetic variation at the *CYP1A2* locus provides a fundamental molecular explanation for the wide spectrum of caffeine metabolic rates observed in the human population.

Beyond genetic variation within the *CYP1A2* gene itself, its expression is critically regulated by the aryl hydrocarbon receptor (AHR) pathway. The AHR is a ligand-activated transcription factor that resides in the cytoplasm in an inactive complex with several chaperons [95]. Upon binding to a diverse array of environmental ligands, the AHR translocate to the nucleus, dimerizes with the AHR nuclear translocator (ARNT) to form an active heterodimer, and binds to xenobiotic response elements (XREs) in the promoter regions of its target genes [72]. This action initiates the transcription of a battery of genes encoding drug-metabolizing enzymes including CYP1A2. Thus, genetic polymorphisms in the *AHR* gene can modulate the sensitivity of this inductive response, adding another layer of interindividual variability. The combined genetic landscape of both *CYP1A2* and *AHR* ultimately determines an individual's baseline and inducible metabolic phenotype.

While these candidate gene studies have been invaluable, recent genome-wide association studies (GWAS) provide a powerful, hypothesis-free approach to uncover novel genetic loci influencing complex traits like caffeine consumption and metabolism. By scanning millions of genetic markers across the entire genome in large cohorts, GWAS can identify regions statistically associated with these phenotypes, often revealing unexpected biological pathways. Large-scale meta-analyses of GWAS on caffeine-related traits have consistently confirmed the paramount importance of the *CYP1A2* (encoding the major enzyme in caffeine metabolism) and *AHR* (encoding the key transcriptional regulator of *CYP1A2* expression) loci, solidifying their central role [31, 34, 110]. Meanwhile, these studies have identified other significant associations with additional genes including *POR* and *MLXIP*, painting a more complex picture of the genetic architecture. (Table 3).

**Table 3:** Filtered GWAS results of caffeine tolerance

SNPS	Region	Mapped Gene	SNP-risk Allele	P-Value	BETA	95% CI
rs6968865	7p21.1	*AHR*	rs6968865-T	2 × 10^–11^	0.26	[0.15–0.36] cups per day increase
rs4410790	7p21.1	*AHR*	rs4410790-T	3 × 10^–17^	0.1	[0.08–0.12] unit decrease
rs6968554	7p21.1	*AHR*	rs6968554-A	7 × 10^–15^	0.2	[0.14–0.26] unit decrease
rs2472297	15q24.1	*CYP1A1—CYP1A2*	rs2472297-T	5 × 10^–14^	0.31	[0.17–0.44] cups per day increase
rs2470893	15q24.1	*CYP1A1—CYP1A2*	rs2470893-T	5 × 10^–14^	0.12	[0.08–0.16] mg/day increase
rs7800944	7q11.23	*MLXIPL*	rs7800944-T	3 × 10^–11^	0.05	[0.030–0.070] unit decrease
rs17685	7q11.23	*POR*	rs17685-A	1 × 10^–9^	0.08	[0.060–0.100] unit increase

**Fig. 3:**
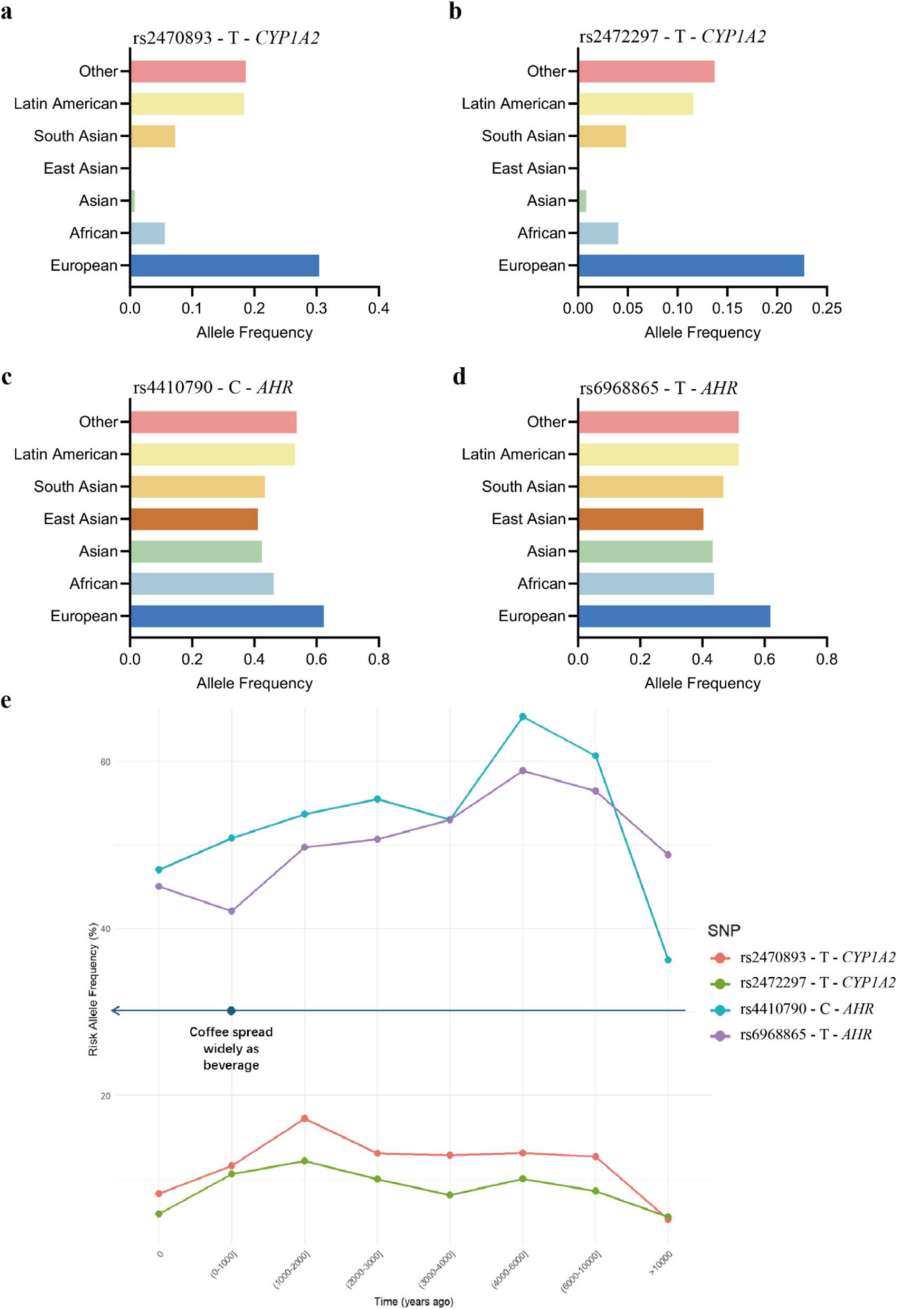
Allele frequency distribution of caffeine-related variants across populations and time. **a-d** Population-level risk allele frequencies for four caffeine-associated SNPs, adapted from data in Table 4; **e** Temporal dynamics of the risk allele frequencies of the four variants across ancient human populations over a range of time periods. Each line represents a different SNP, with associated historical cultural markers indicated on the timeline

To systematically elucidate the genetic basis of caffeine metabolism, we extracted top-hit SNPs associated with caffeine consumption (p < 10⁻⁹) from the NHGRI-EBI GWAS Catalog (accessed 20 May 2025; https://www.ebi.ac.uk/gwas/efotraits/EFO_0004330). From these, we focused on SNPs mapping to *CYP1A2* and *AHR* given their well-established biological roles. The aggregation of SNP data from diverse populations further enables the examination of allele frequency distributions on a global scale. We retrieved population-level allele frequencies for four representative high-significance SNPs—rs2472297 and rs2470893 (mapping to *CYP1A2*), and rs4410790 and rs6968865 (mapping to *AHR*)—from the ALFA project [96]. Our analysis reveals striking interpopulation heterogeneity: alleles associated with enhanced caffeine metabolism (e.g., rs2472297-T and rs2470893-T) are most frequent in European populations and least frequent in East Asian populations. Similarly, AHR variants exhibit frequency differences across continental groups, (Table 4) (Fig. 3a-d).

**Table 4:** Population-specific allele frequencies of key caffeine metabolism-associated SNPs from ALFA (Release Version: 20250407153717)

Population	Variant and risk allele			
	rs2470893—T	rs2472297—T	rs4410790—C	rs6968865—T
European	0.299381	0.223383	0.620144	0.61814
African	0.0524	0.03404	0.4639	0.4367
Latin American	0.1755	0.1132	0.526	0.516
Asian	0.0003	0.0006	0.4373	0.4330
South Asian	0.0676	0.0446	0.4291	0.467
East Asian	0.0000	0.0008	0.4111	0.4028
Other	0.18991	0.13953	0.5619	0.5161

This non-random geographical distribution of caffeine-metabolism alleles is a robust signature that suggests past evolutionary pressures have shaped the current genetic landscape. The persistence of these frequency raises a critical question: when and why did these allelic differences arise? To address this, we need to move beyond the spatial dimension of modern populations and interrogate the temporal dimension through the analysis of ancient DNA.

Our analysis of genome-wide data from a panel of ancient individuals revealed an interesting temporal pattern for the key rapid-metabolism allele in *CYP1A2* (rs2472297-T and rs2470893-T). The frequency of both alleles witnessed a marked and sustained increase around 1000 years ago (Fig. 3e). However, alleles in the *AHR* gene region, which has a more complex role in responding to diverse environmental toxins, exhibited greater variability and less consistent temporal patterns in our ancient DNA dataset.

Notably, the insights from GWAS must be interpreted in the context of its methodological limitations. The genetic analysis presented here focused on a limited set of GWAS-identified SNPs mapped to the *CYP1A2* and *AHR* genes, allowing us to concentrate on variants within well-established biological pathways to obtain more interpretable results. While informative, this approach necessarily overlooks the broader genetic architecture of caffeine tolerance, which is undoubtedly influenced by a more complex and polygenic array of variants across the genome.

These genetic and evolutionary findings paint a compelling picture: the wide variation in caffeine tolerance we see today could be the product of deep evolutionary history, shaped by ancient selective pressures acting on our detoxification machinery. However, these genetic adaptations did not unfold in a vacuum; we believe that they intersected with a parallel history of cultural and global exchange. To fully understand caffeine consumption patterns, we need to now turn to the cultural and historical trajectories that brought coffee, tea, and other caffeine sources from their regional origins to global prominence, interacting with the human genetic landscape in complex and meaningful ways.

### Evolution of caffeine tolerance genes and cultures

The genetic and evolutionary adaptations that shape human responses to caffeine are mirrored in the historical trajectory of its consumption. The spread of consumption habits across cultures reveals how biological, social, and economic factors intertwined to shape one of the world’s most widely used psychoactive substances. This section traces the historical pathways of caffeine and highlights key events that influenced the development and diversification of caffeine tolerance in human populations, thereby contextualizing its contemporary patterns of use.

Coffee, derived from the seeds of plants belonging to the genus Coffea, primarily *Coffea arabica* and *Coffea canephora* var. *robusta*, has a rich history rooted in the ancient cultures of Africa and the Middle East. *Coffea arabica* has been proven indigenous to the highlands of Ethiopia, southeastern Sudan, and northern Kenya. Early uses of the coffee plant by indigenous African communities involved consuming the leaves, fruits, and seeds as food [120].

The transmission of coffee from Africa to Arabia likely occurred in pre-Islamic times. Initially consumed in solid form as compressed bars—similar to modern chocolate bars—coffee was used by Ethiopian crusaders as a portable ration during military campaigns as early as the sixth century AD [113]. By the mid-fifteenth century, coffee brewing as a beverage emerged in Yemen among Sufi practitioners, who used it to sustain concentration during long religious rituals [120]. The first coffee houses appeared in Mecca in 1470, spreading to Cairo, Damascus, and Istanbul by the mid-sixteenth century [105]. In the mid-seventeenth century, coffee consumption expanded beyond the Middle East aided by European colonial and trade networks. The Dutch and French played key roles in its global dissemination: coffee was introduced to Suriname in 1718, and shortly after to French Guiana and Brazil. Similarly, French naval brought coffee to Martinique in 1720, enabling its spread throughout the Caribbean [120].

While coffee represents a major historical vector of caffeine consumption, other plants such as cacao have also been significant, particularly in different cultural and geographical contexts. Cacao (Theobroma cacao), native to Mesoamerica, was consumed by ancient Maya and Aztec civilizations long before European contact. Archaeological evidence from the Classic (AD 250–900) and Postclassic (AD 900–1500) periods includes preserved cacao beans, charcoal fragments, and iconographic representations on ceramics and sculptures [98].

The historical dissemination of caffeine sources, particularly the rapid global spread of coffee from the 6th to sixteenth century, presents a compelling cultural parallel to the genetic changes uncovered in our ancient DNA analysis. Our genetic data indicates a rise in the frequency of alleles associated with enhanced caffeine metabolism—an event tentatively dated to approximately 1,000 years before present. While the precise selective drivers remain complex and were likely multifaceted, the temporal coincidence between this genetic shift and the documented rise in caffeine availability is interesting. The widespread adoption of coffee drinking, first within the Arab world and later globally, may have begun to exert a novel selective pressure—or, more plausibly, amplified an existing one—favoring individuals with genetic predispositions for efficient caffeine clearance. This potential gene-culture co-evolutionary interaction provides a possible context for interpreting the observed allele frequency changes. It suggests that the biological tolerance we see today was not only shaped by ancient environmental pressures but may have been significantly reinforced by historically more recent caffeine consumption practices.

It is important to acknowledge a limitation inherent in our study design regarding the selection of genetic variants for ancient DNA analysis. Our investigation was constrained to the four GWAS-significant SNPs (rs2472297, rs2470893, rs4410790, rs6968865) that formed the initial focus of our broader project. This approach, while powerful for identifying intergenic signals of selection across populations and time, does not encompass the full spectrum of functional variation known to influence caffeine metabolism. Meanwhile, inferring evolutionary causation from temporal coincidence presents a significant challenge. Although we observe an intriguing alignment between the rise in caffeine metabolism alleles and the historical spread of coffee culture, we cannot definitively attribute this selection pressure to caffeine exposure itself. The CYP1A2 enzyme metabolizes a wide array of xenobiotics, and the selective forces driving the frequency changes in these alleles may have been related to other dietary, environmental, or pathological factors entirely. Therefore, the proposed link to caffeine consumption remains a plausible but unproven hypothesis.

### Non-genetic modulators of caffeine metabolism: lifestyle, physiology and medication

While genetic polymorphisms establish the foundational blueprint for an individual's caffeine metabolic capacity, as detailed in previous sections, the ultimate phenotypic expression is dynamically shaped by a variety of extrinsic factors [47]. Lifestyle choices, physiological states, and medication use can profoundly induce or inhibit CYP1A2 activity, leading to clinically significant shifts in caffeine pharmacokinetics and response. This section synthesizes evidence on three major modulators: tobacco smoking, pregnancy, and pharmaceutical agents.

Tobacco smoke is one of the most well-characterized and powerful environmental inducers of CYP1A2 activity. The induction of *CYP1A2* by tobacco smoke is primarily mediated through the *AHR* pathway described earlier. Polycyclic aromatic hydrocarbons (PAHs) in smoke serve as potent ligands that activate this receptor, leading to a significant upregulation of *CYP1A2* gene expression and a consequent increase in enzyme activity [72, 78].

This induction leads to significantly enhanced caffeine clearance in smokers. Pharmacokinetic studies consistently demonstrate that smokers exhibit a ~ 40–65% increase in caffeine clearance compared to non-smokers. For instance, the mean body clearance of caffeine was reported to be 2.0 ± 0.8 mL·kg⁻^1^·min⁻^1^ in smokers versus 1.5 ± 0.7 mL·kg⁻^1^·min⁻^1^ in non-smokers [14], while the elimination half-life (t½) decreased from 6.0 h to 3.5 h [94]. This accelerated metabolism is specifically mediated by *CYP1A2* induction, as cigarette smoking does not significantly alter the activity of other caffeine-metabolizing enzymes such as xanthine oxidase or N-acetyltransferase-2. This pharmacokinetic shift provides a biological mechanism for the observed positive correlation between smoking and high coffee consumption. This correlation has been attributed to both shared genetic factors (rg ≈ 0.47) and the direct environmental induction of caffeine metabolism by smoking (re ≈ 0.30). Consequently, smoking status should be considered a critical modifier when evaluating caffeine consumption patterns and its associated health effects.

In contrast to the inducing effects of smoking, pregnancy represents a profound physiological state of CYP1A2 suppression. This significant attenuation of enzyme activity is driven by the complex hormonal profile of gestation, particularly elevated levels of progesterone and estrogen, which are believed to directly interfere with enzyme function or its transcriptional regulation [58].

This suppression leads to a marked and progressive decline in caffeine clearance throughout pregnancy. Pharmacokinetic studies demonstrate that CYP1A2 activity is significantly reduced by ~ 33% in the first trimester, ~ 48% in the second trimester, and ~ 65% in the third trimester compared to the postpartum period [116]. Consequently, the elimination half-life of caffeine is drastically prolonged, increasing from an average of 3 h in non-pregnant women to as long as 10.5 h during the final weeks of gestation [66]. This impaired clearance results in substantially elevated plasma caffeine concentrations, thereby increasing fetal exposure across the placenta. Beyond increasing the risk of restricted fetal growth, in utero caffeine exposure may disrupt key developmental processes and possibly induce epigenetic changes. These alterations have been linked to an elevated susceptibility to cardiometabolic defects in the offspring and may increase the risk of adult-onset diseases in subsequent generations [99].

A wide array of commonly prescribed medications also acts as potent inhibitors of CYP1A2, leading to clinically significant pharmacokinetic interactions with caffeine. The foundation for these interactions lies in caffeine's status as a low-clearance substrate with relatively low affinity (Km ≈ 500 µmol/L) for CYP1A2, making its metabolism highly susceptible to competitive inhibition by other drugs that share the enzyme [48]. Ethinylestradiol-containing combination oral contraceptives reduced caffeine clearance by approximately 55% after one treatment cycle [8]. Antimycotic agents decrease caffeine clearance—oral terbinafine 500 mg by ~ 21% and fluconazole by ~ 25% [122, 130]. Fluoroquinolone antibiotics also significantly inhibit caffeine metabolism [109]. For example, enoxacin at a dosage of 800 mg per day reduces caffeine clearance by approximately 80% [65]. Notably, the central muscle relaxant idrocilamide profoundly impaired caffeine biotransformation, resulting in an approximately ninefold prolongation of caffeine half-life and the emergence of caffeine-related adverse effects including insomnia, anxiety, hyperactivity, confusion and delirium [17]. (Table 5).

**Table 5:** Modulation of CYP1A2 Activity and Caffeine Metabolism by Key Extrinsic Factors

Modulator	Effect on CYP1A2	Magnitude of Change	Key Mechanisms
Tobacco Smoke	Induction	Clearance ↑40–65% t₁/₂ ↓ ~ 40%	PAHs activate AHR, upregulating *CYP1A2* gene expression
Pregnancy	Inhibition	CYP1A2 Activity ↓ ~ 50% t₁/₂ ↑ ~ 250%	Elevated progesterone and estrogen suppress enzyme activity/expression
Contraceptives	Inhibition	Clearance↓ ~ 55%	Competitive inhibition of CYP1A2 enzyme activity
Fluconazole		Clearance↓ ~ 25%	
Enoxacin		Clearance↓ ~ 80%	
Idrocilamide		t₁/₂ ↑up to 9x	

These interactions underscore the critical importance of considering concomitant medication use when evaluating caffeine consumption, as inhibition of CYP1A2 can mimic a slow metabolizer phenotype, elevating plasma caffeine concentrations and increasing the risk of toxicity, even in individuals with a genetic predisposition for rapid metabolism.

### Health implications of caffeine

Coffee is a chemically rich beverage composed of numerous bioactive constituents, each contributing distinct physiological effects [104]. The net health impact of coffee consumption is determined by the balance of these components, which is influenced by dose and preparation methods.

Caffeine is the primary compound in coffee with highly dose-dependent effects. At low to moderate doses (100–200 mg, equivalent to ~ 1–2 standard cups), it reliably enhances arousal, alertness, concentration, and performance on cognitive and motor tasks. At high doses (> 500 mg), it can induce adverse effects including nervousness, restlessness, agitation, tremors, nausea, and tachycardia. Acute toxicity is rare but severe, with lethal overdoses typically requiring ingestion in excess of 5 g, leading to arrhythmias, convulsions, and coma [61, 62]. Epidemiological studies have showed that dietary caffeine intake reliably impairs night sleep quality by antagonizing adenosine receptors. Notably, consumption of caffeine equivalent to one or two double-espressos—even up to 16 h before bedtime—can induce measurable changes in sleep EEG patterns, indicative of more superficial sleep [28].

In addition, coffee is the foremost dietary source of some polyphenols with an average 180 mL cup providing approximately 400 mg of these antioxidant compounds [50, 121]. The most abundant and biologically significant subclass is the chlorogenic acids (CGAs), which constitute 70–350 mg per 200 mL cup depending on bean variety and roast [20, 29]. CGAs are not only antioxidants but also potent modulators of cellular defense pathways [88]. They activate the transcription factor Nrf2 (Nuclear factor erythroid-2-related factor 2), the master regulator of the antioxidant response, leading to the upregulated expression of cytoprotective enzymes [106]. Furthermore, CGAs improve endothelial function by enhancing nitric oxide bioavailability and exert anti-inflammatory effects by inhibiting the production of pro-inflammatory cytokines such as TNF-α and IL-6 [70, 111]. Through these mechanisms, CGAs are implicated in the protective effects of coffee against diseases such as atherosclerosis by inhibiting the oxidation of low-density lipoprotein (LDL) cholesterol in the plasma and below the endothelial surface [86, 129].

In contrast to the largely beneficial polyphenols, the diterpene compounds cafestol and kahweol represent a notable exception in coffee's health profile [23]. A typical bean of C. arabica contains about 0.3–0.7% cafestol and 0.1–0.3% kahweol by weight, producing cafestol and kahweol with individual concentrations ranging from 0.1 to 7 mg/ml in coffee [82]. These compounds, found in the oily fraction of coffee, are among the most potent dietary elevators of serum cholesterol levels in humans. They act by downregulating the hepatic expression of LDL receptors, impairing the clearance of LDL-cholesterol from the bloodstream. Meta-analyses of randomized controlled trials indicate that consumption of boiled coffee dose-dependently is associated with higher serum total and LDL cholesterol concentrations, while the consumption of filtered coffee results in slight increase in serum cholesterol. This discrepancy in findings is primarily because filtered coffee contains only minor amounts of diterpenes [59, 71].

The diverse bioactive compounds in coffee create a complex profile of health effects, acting in multiple and sometimes opposing ways [45]. Epidemiological research has consistently linked coffee consumption to a reduced risk of several major diseases, including type 2 diabetes through improved glucose tolerance [119], neurodegenerative disorders such as Parkinson's disease [126], and certain cancers [22]. Regular coffee intake is associated with lower systemic inflammation, as evidenced by reduced plasma concentrations of inflammatory markers [76], and may also support metabolic health by reducing body fat [84].

However, the most extensive and compelling body of evidence, as well as the most significant public health implications, centers on its relationship with cardiovascular health [117]. The cardiovascular health effects of coffee consumption represent a balance of beneficial and potentially adverse components, where polyphenols confer protective properties, whereas diterpenes and caffeine may raise lipid levels and influence endothelial function, respectively.

Large-scale meta-analyses, encompassing data from over 1.2 million participants, consistently demonstrate a nonlinear association between coffee consumption and cardiovascular disease (CVD) risk [37]. This relationship is frequently characterized as a "J-shaped" curve, where moderate consumption is associated with the lowest risk. The lowest risk is typically observed at 3 to 5 cups per day, with this level of intake associated with an approximate 15% reduction in CVD risk compared to non-consumption [102]. In a study of a Greek population, moderate coffee drinking (< 300 ml/day) reduced the risk of developing acute coronary syndromes 31% relative to no consumption [93]. With regard to stroke, results from women followed during 24 years [75] showed that habitually consuming 2–4 cups of coffee per day was associated with a 20% lower risk. Moreover, a 15-year study of over 40,000 postmenopausal US women found that coffee consumption was associated with altered CVD mortality, with fully adjusted hazard ratios of 0.76 (1–3 cups/day), 0.81 (4–5 cups/day), and 0.87 (≥ 6 cups/day), indicating that moderate consumption may achieve the optimal protective effect [4]. However, it is important to note that epidemiological evidence alone cannot establish a causal relationship between coffee intake and CVD. This is especially true given that CVD is a multifactorial condition and it is impossible to fully disentangle the effect of coffee from other lifestyle covariates that accompany its consumption [37].

In addition to the amount of coffee consumed, the method of coffee preparation is also a paramount determinant of its cardiovascular effects, primarily due to the differential content of the cholesterol-elevating diterpenes. Meta-analyses of randomized controlled trials confirm that heavy drinking of boiled coffee (> 600 ml/day) is very detrimental to heart health [53]. In a Swedish case–control study, men who consumed boiled coffee exhibited a 1.4-fold higher risk of first nonfatal myocardial infarction compared to those who drank filtered coffee [51].

A common concern regarding coffee and cardiovascular health is its potential to induce hypertension (HTN), a major risk factor for CVD. Acute studies show that caffeine can cause a transient rise in blood pressure, particularly in non-habitual consumers [35]. However, long-term epidemiological data does not support a sustained hypertensive effect from habitual consumption. A comprehensive meta-analysis found no clinically important effects of long-term coffee intake on blood pressure or the risk of developing hypertension [35, 124]. Another long-standing notion that caffeine consumption provokes cardiac arrhythmias has been substantially challenged by a recent, large-scale study, where population-based community cohort study found that greater amounts of habitual coffee consumption were associated with a lower risk of arrhythmia [64].

**Fig. 4:**
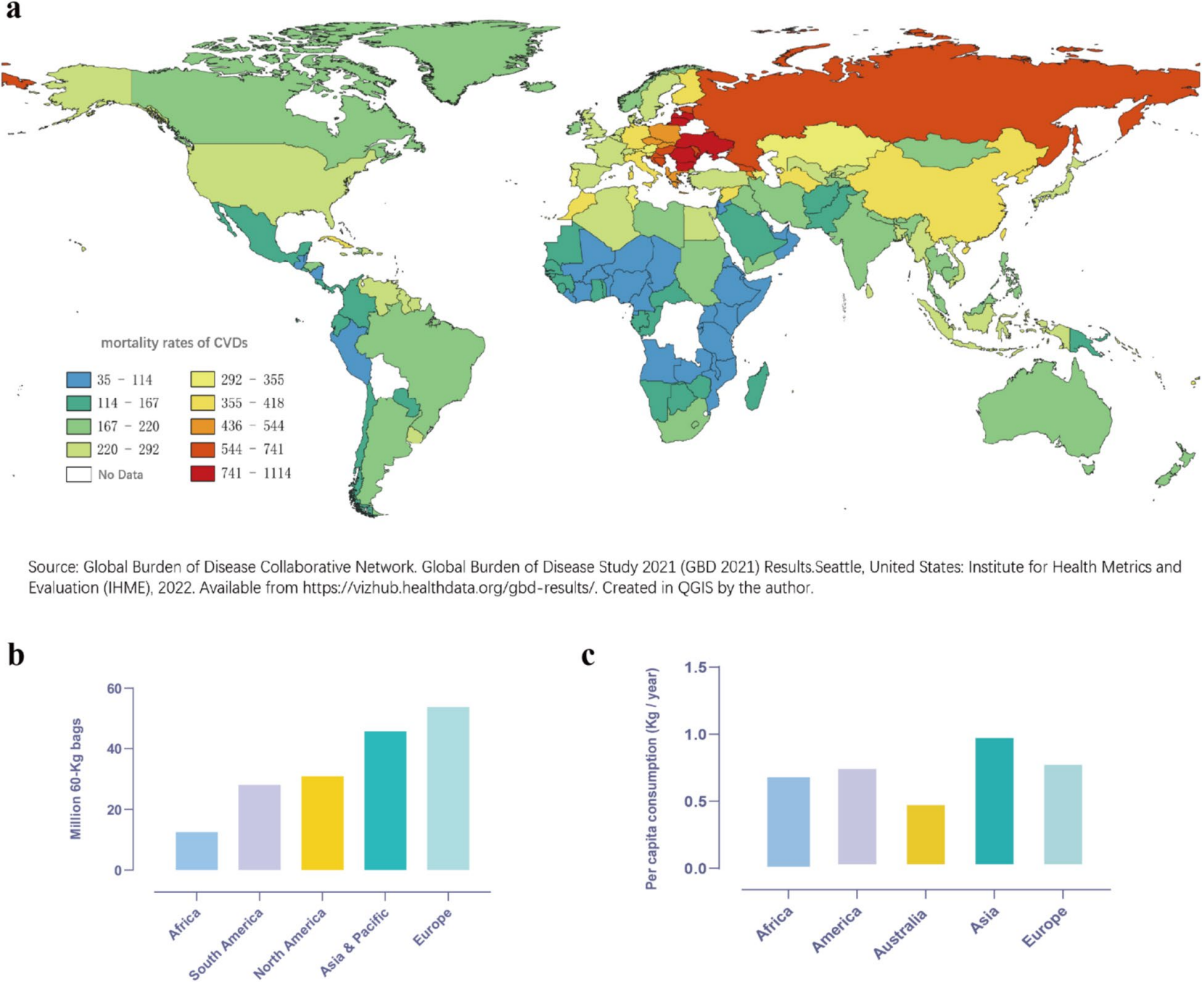
Association between coffee intake and cardiovascular disease mortality. **a** Global map of cardiovascular disease mortality in 2021, data sourced from Global Burden of Disease Study 2021 Results; **b** Coffee consumption in 2023 in different regions, data sourced from International Coffee Organization; **c** Tea consumption in 2024 in different regions, data sourced from Tea Statistics by Country, Tea Consumer Behavior and Facts (2025)

It is worth noting that these population-level associations may mask considerable interindividual variability. We believe that a proportion of this variability can be partly explained by genetic differences in caffeine metabolism. This gene-diet interaction partly modulates the relationship between coffee consumption and cardiovascular outcomes. The key genetic determinants discussed in previous sections—such as the slow-metabolism-associated alleles of *CYP1A2* (e.g., rs2069514-A, rs2470893-C) and the rapid-metabolism-associated alleles (e.g., rs762551*-A*, rs2472297-T)—enable the distinction between metabolizer phenotypes. Epidemiological data from the Institute for Health Metrics and Evaluation (IHME) reveal intriguing population-level patterns (Fig. 4a). Populations in Europe and North America, where rapid-metabolizer alleles are more frequent, generally experience lower cardiovascular mortality alongside high coffee consumption. In contrast, some Asian populations exhibit higher cardiovascular mortality despite increasing coffee intake (Fig. 4b), a trend that aligns with their higher prevalence of slow-metabolizing *CYP1A2* alleles. This suggests that slow metabolizers, exposed to higher systemic caffeine concentrations per unit consumed, may not derive the same cardiovascular benefits from coffee and could potentially face elevated risks. However, it is crucial to note that a causal link has not yet been established. The addition of tea consumption data (Fig. 4c) provides further context that Asian and European are relatively high tea-consuming populations [44]. This pattern challenges a simple, direct correlation between coffee consumption and CVD benefit and instead suggests that the relationship is likely confounded and modified by other factors.

Apart from cardiovascular effects, this gene-diet interaction of caffeine intake is powerfully demonstrated in the context of renal health. A recent prospective study followed young adults with stage 1 hypertension for a median of 7.5 years and found that heavy coffee consumption (> 3 cups/day) was associated with significantly increased risks of albuminuria, hyperfiltration, and hypertension exclusively among slow metabolizers (AC/CC genotypes, *CYP1A2* at rs762551). Crucially, no such associations were observed in fast metabolizers (AA genotype), providing compelling evidence that the adverse renal effects of caffeine are contingent upon an individual's inherited metabolic capacity [79]. Moreover, a study demonstrated that the *CYP1A2* rs762551 polymorphism critically modifies the association between coffee consumption and body mass index (BMI), with rapid metabolizers (AA genotype) not only consuming more coffee but also more likely to experience its appetite-suppressing effects [42]. These findings underscore that the metabolic benefits of caffeine may not be universal but are concentrated in individuals with the rapid metabolizer phenotype, offering a compelling rationale for incorporating pharmacogenetic profiling into personalized nutritional strategies for obesity.

These findings highlight the need for a personalized approach to coffee consumption that incorporates genetic background [127], brewing method [15], and concomitant dietary habits [91]. A nuanced understanding of how genetics and preparation modulate coffee’s physiological effects is essential for assessing its role in cardiovascular and neurological health.

Beyond its widespread dietary consumption and associated population health effects, caffeine is also a molecule of significant pharmacological interest. Its primary and well-established use is as a first-line treatment for apnea of prematurity in infants, where it stimulates breathing [1]. In adults, caffeine is an effective adjuvant therapy for pain relief, particularly for migraines and tension-type headaches, with doses of ≥ 100 mg shown to enhance the efficacy of analgesics [9, 74]. Recent research highlighted that low-dose caffeine (3–5 mg/kg body weight) may reduce neurodegenerative risk by antagonizing A2A receptors [101].

However, the therapeutic potential of caffeine is often constrained by its rapid and widespread distribution, short elimination half-life, and dose-dependent side effects. To overcome these limitations, nanotechnology-based delivery strategies have emerged as a promising frontier, which aim to enhance bioavailability, enable targeted tissue delivery, and achieve sustained release [103, 108]. A recent preclinical study demonstrated the efficacy of caffeine-loaded chitosan nanoparticles in ameliorating obesity-induced cardiovascular complications in a rat model [54].

### Caffeine and beyond: the distinctive bioactive profile of tea

**Fig. 5:**
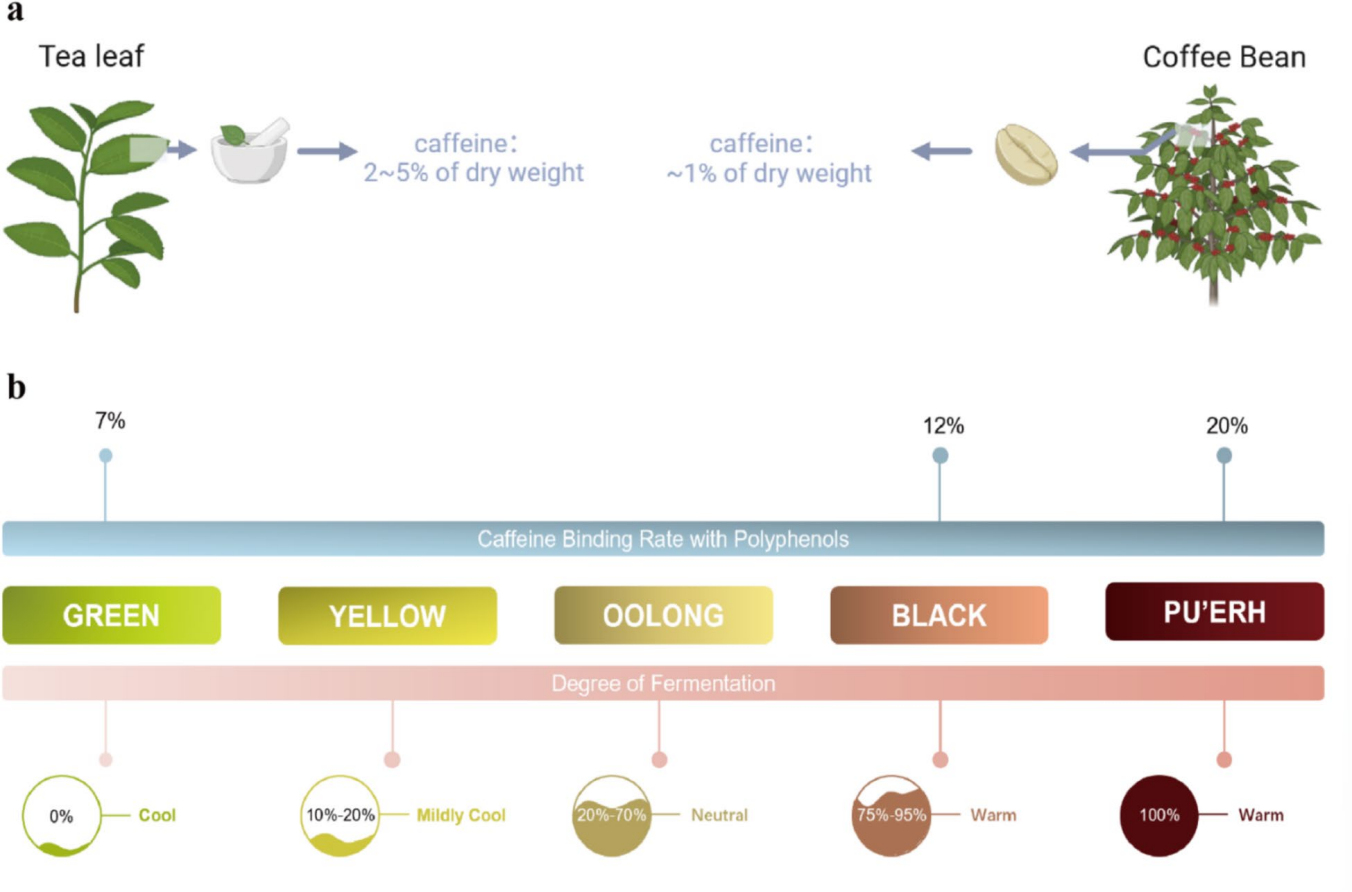
Caffeine content in tea and coffee and polyphenol binding properties of caffeine. **a** Caffeine content in tea leaves and coffee beans by dry weight;** b** Caffeine binding rate with polyphenols and fermentation levels across five major types of tea. Binding rates increase from green tea (7%) to Pu-erh tea (20%), correlating with increasing fermentation degrees and warming properties

Like coffee, tea (*Camellia sinensis*) is a major dietary source of caffeine, regularly consumed by more than two-thirds of the global population [6, 83]. Interestingly, on a dry-weight basis, tea leaves contain a higher proportion of caffeine (2–5%) than coffee beans (1.1–2.2%) [30] (Fig. 5a). Despite this, coffee is generally perceived as a stronger stimulant, and caffeine-related intolerance is more frequently associated with coffee consumption than tea.

This apparent contradiction can be attributed to two main factors. First, the actual caffeine content in a typical beverage serving differs significantly between the two. A standard cup of brewed coffee (200 mL) contains between 60 and 125 mg of caffeine, whereas the same volume of tea provides only about 28–42 mg [27]. Second, tea contains several unique bioactive compounds, such as polyphenols and the amino acid L-theanine that are known to modulate the pharmacological effects of caffeine, potentially mitigating its stimulatory impact.

The actual caffeine content delivered in a cup of tea is influenced by several key factors, primarily the degree of roasting, fermentation, and infusion conditions. During tea processing, oxidative polymerization of polyphenolic compounds enhances their ability to bind with caffeine [26]. This reduces the amount of free, soluble caffeine available in the final brew (Fig. 5b). Consequently, lightly oxidized teas—such as green tea—retain higher levels of bioavailable caffeine compared to heavily oxidized varieties like black or Pu-erh teas [55, 83]. In addition, microbial activity during fermentation also plays a significant role in modulating caffeine levels. Molds such as Aspergillus niger can increase caffeine concentrations in tea, whereas certain yeasts may reduce them [123]. Biochemical studies suggest that microbial metabolism can convert biosynthetic precursors like theophylline into caffeine, leading to higher caffeine content in fermented teas than in fresh leaves [73]. Finally, infusion conditions strongly dictate caffeine extraction. Longer steeping times and higher water temperatures significantly increase caffeine yield. For instance, in typical China green tea, the caffeine content in a 180 mL infusion increases from 23 to 41 mg as the steeping time ranges from 1 to 5 min [27].

Beyond caffeine, tea is particularly rich in polyphenols, which represent the most biologically active group of its constituents. Polyphenols account for up to 30% of the dry weight of green tea leaves, of which the major catechins are (–)-epigallocatechin-3-gallate (EGCG), (–)-epigallocatechin (EGC), (–)-epicatechin (EC), (–)-epicatechin gallate (ECG), and (+)-catechin (C) [25, 43, 73]. Comparative analyses show that green tea contains markedly higher levels of catechins (67 ± 11 mg/g dry matter) than black tea (15 ± 5 mg/g), reflecting the impact of fermentation [63]. During black tea manufacture, monomeric flavan-3-ols undergo polyphenol oxidase–dependent oxidative polymerization, forming theaflavins, bisflavanols and other oligomers [73]. Thus, while green tea is enriched in catechins, black tea contains more complex polyphenolic oligomers [7].

The biological activities of tea polyphenols are diverse, encompassing strong antioxidative effects [24, 90]. Catechins such as EGCG are particularly well studied for their cancer-preventive properties [128]. Collectively, these properties suggest that catechins contribute not only to the antioxidant potential of tea but also to its long-term health benefits through modulation of cellular signaling and detoxification pathways.

Another distinctive bioactive component of tea is L-theanine, a non-proteinogenic amino acid found almost exclusively in *Camellia sinensis*. L-theanine constitutes about 1–2% of the dry weight of tea, corresponding to roughly 25–60 mg per 200 mL serving [18]. It is structurally analogous to glutamic acid and readily crosses the blood–brain barrier, reaching peak levels within 30 min and sustaining presence for several hours [87].

Mechanistically, L-theanine modulates neurotransmitter systems in the central nervous system. It increases dopamine release [125], enhances GABA concentrations, inhibits glutamate reuptake, and blocks glutamate receptors in the hippocampus [60]. These actions collectively produce a calming effect and lower blood pressure [39]. Importantly, L-theanine appears to antagonize the stimulatory effects of caffeine, yielding a more balanced psychophysiological response. This interaction provides a plausible explanation for why tea consumption often results in a state of relaxed alertness, contrasting with the sharper stimulation associated with coffee.

Taken together, tea contains a wide array of bioactive compounds beyond caffeine (Table 6). The high content of polyphenols, especially catechins such as EGCG, underpins much of tea’s antioxidative properties, albeit with limited oral bioavailability. In parallel, L-theanine contributes a unique neurophysiological profile by modulating neurotransmission and counterbalancing caffeine’s stimulatory action. These constituents provide a scientific rationale for the distinctive cognitive and health effects of tea compared to coffee.

**Table 6:** Bioactive compounds in tea and coffee

Beverage	Component	Typical content	Primary effects
Coffee	Caffeine	1.1–2.2% (dry weight)	Central nervous system stimulation; enhanced alertness
	Polyphenols	~ 400 mg per 180 mL serving	Antioxidants; improve endothelial function; anti-inflammatory; inhibit LDL oxidation
	Diterpenes	0.1–0.7% (dry weight)	Elevators of serum cholesterol
Tea	Caffeine	2–5% (dry weight)	Central nervous system stimulation; enhanced alertness
	Catechins	30–42% (dry weight)	Antioxidants; anticarcinogenic; cardiovascular protection
	L-theanine	25–60 mg per 200 mL serving	Calming effect; counteracts caffeine’s stimulation

## Discussion

The extensive individual variability in caffeine sensitivity and tolerance presents a fascinating puzzle at the intersection of genetics, evolution, and lifestyle. This review has synthesized evidence demonstrating that this variability is not a random phenomenon but rather the predictable outcome of a complex interplay between an individual's genetic blueprint, evolutionary history, and environmental exposures.

We have delineated the journey of caffeine from consumption to clearance, establishing that CYP1A2-mediated metabolism is the primary determinant of subsequent physiological effects. The wide interindividual variability in CYP1A2 activity emerges as the central axis upon which caffeine tolerance revolves. This variability is rooted in genetic polymorphisms within the *CYP1A2* gene and its regulator, *AHR*, which create a population continuum from slow to rapid metabolizers. Notably, our integration of ancient DNA analysis suggests that the genetic landscape underlying this metabolism was shaped by evolutionary pressures, potentially tied to dietary shifts during the spread of coffee. It is plausible that the cultural adoption of coffee and tea created a novel selective environment where efficient caffeine metabolizers experienced a relative advantage, potentially amplifying and shaping the very genetic differences we observe today.

Critically, this genetic predisposition is not deterministic. We have highlighted how potent environmental and physiological modulators—such as tobacco smoke, pregnancy and medication—can dramatically alter CYP1A2 activity. This dynamic modulation explains why an individual's caffeine response can change over their lifetime and must be contextualized within their current health and lifestyle status.

Synthesizing the information outlined above, the health consequences of caffeine intake are shaped by the integrated effects of this gene-environment interaction. The benefits and adverse effects of prolonged caffeine exposure are balanced differently in each individual, depending largely on their metabolic phenotypes. Our findings elucidate the epidemiological observation that populations with a higher prevalence of slow-metabolizer alleles may not derive the same cardiovascular benefits from high coffee consumption as populations with a higher frequency of rapid-metabolizer alleles.

In conclusion, our analysis demonstrates that individual responses to caffeine arise from a complex interplay of *CYP1A2* and *AHR* genetic variants, modifiable environmental influences, and population-specific allele frequencies shaped by evolutionary pressures. Translating these findings into clinical practice requires a structured framework: (1) Assess metabolic phenotypes by incorporating individual genetic profiling to distinguish slow from rapid metabolizers; (2) Tailor regional guidelines to account for population-specific genetic architectures; (3) Evaluate concomitant medications and physiological status to identify factors altering CYP1A2 enzyme activity or influencing caffeine metabolism through alternative pathways.

Complementing the genetic and physiological framework, personalized caffeine recommendations should integrate practical consumption patterns. Beverage preparation methods critically influence health outcomes: for example, filtered coffee removes diterpenes that elevate cholesterol levels, thereby reducing cardiovascular risks inherent to unfiltered preparations. Individuals with pre-existing cardiovascular risk factors are thus advised to prioritize filtered coffee to retain beneficial bioactive compounds while minimizing cardiovascular strain. Additionally, individual tolerance varies markedly across caffeine sources. Those sensitive to coffee's effects may benefit from switching to tea, whose unique phytochemical profile modulates caffeine's stimulatory properties, potentially eliciting a gentler physiological response. These practical considerations provide actionable guidance that synergizes with pharmacogenetic insights, enabling a comprehensive approach to personalized caffeine consumption.

By transcending uniform recommendations to embrace this multidimensional perspective, we can optimize caffeine's benefits while mitigating risks. This strategy paves the way for nutrition recommendations that integrate genetic predispositions, current health metrics, and environmental exposures.

While this framework offers a tangible path toward personalizing caffeine intake, it is important to acknowledge that our synthesis has its boundaries. The conclusions of this should be considered in light of the methodological limitations discussed in preceding sections, particularly regarding the scope of genetic analysis and evolutionary interpretation. These point to a direction for future research employing more comprehensive sequencing and functional analyses to disentangle the specific selective agents that have shaped human metabolic evolution. Future research should also prioritize large-scale, longitudinal studies that simultaneously genotype participants, quantify caffeine intake, and rigorously assess health outcomes. This will allow for the development of integrated risk models that account for genetic landscape, metabolic capacity, and lifestyle factors. Furthermore, exploring the interaction between caffeine metabolism and the gut microbiome represents a promising new frontier.

## Data Availability

Not applicable.

## Abbreviations

GWAS: Genome-wide associated studies
SNP: Single nucleotide polymorphism
CYP450: Cytochrome P450 enzyme family
GABA: Gamma-aminobutyric acid
